# A formal statechart model of immediate neonatal adaptation guidelines

**DOI:** 10.1016/j.heliyon.2025.e42784

**Published:** 2025-02-19

**Authors:** Edgar Hernando Sepúlveda-Oviedo, Leonardo Enrique Bermeo Clavijo, Luis Carlos Méndez–Córdoba

**Affiliations:** a LAAS-CNRS, Université Fédérale de Toulouse, CNRS, UPS, Toulouse, France; b Universidad Nacional de Colombia, Sede Bogotá, Facultad de Medicina, Departamento de Pediatría, Bogotá, Colombia; c Universidad Nacional de Colombia, Sede Bogotá, Facultad de Ingeniería, Departamento de Ingeniería Eléctrica y Electrónica, Bogotá, Colombia

**Keywords:** Discrete-event medical models, Cardiovascular and respiratory system, Physiologically-based cord clamping, Umbilical cord clamping

## Abstract

Research highlights the importance of applying physiological criteria for optimal umbilical cord clamping, underlining its lasting advantages. In response, the Division of Pediatrics, Perinatology, and Neonatology at the National University of Colombia has pioneered the Immediate Neonatal Adaptation Guideline, focusing on Physiologically-based Cord Clamping. This study has two main objectives: The first is to represent the medical guideline through a statechart model to enhance clarity and detail. Secondly, to evaluate the effectiveness of statechart models in depicting medical guidelines for educational and training purposes within a human-centric framework. In this study, a group of medical professionals and engineers designed the statechart model for the Immediate Neonatal Adaptation guideline through a progressive refinement method. The model comprises 20 states, 38 events, and 4 superstates, offering clear visual language for its evaluation by an interdisciplinary panel of engineers and health professionals. This visual representation facilitates a more explicit identification of patient states, criteria, and clinical indicators involved in the procedure. Feedback indicates general satisfaction with the *Versatility, Usability, Scalability* and *Moderate visual complexity* of the model.

## Highlights

• Formalizes the Immediate Neonatal Adaptation Guideline using statecharts for clarity.
• Proposes a human-centered educational tool for representing medical guidelines.
• Introduces a systematic tool for exploring various medical scenarios.
• Creates a visual language for interdisciplinary collaboration in medical modeling.
• Demonstrates the adaptability and scalability of statecharts in medical guidelines.

## Graphical abstract

**Figure:**
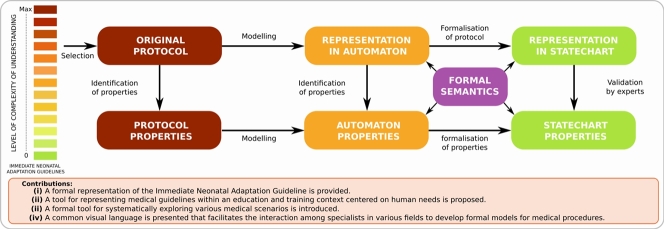

## 1 Introduction

Transitioning from time-driven neonatal care to evidence-based and physiologically aligned practices necessitates the creation of new clinical guidelines and the implementation of advanced approaches for their representation and dissemination [1], [2], [3], [4], [5], [6], [7]. The Immediate Neonatal Adaptation (INA) guideline, formulated by the Division of Perinatal Pediatrics, Perinatology, and Neonatology at the National University of Colombia,^1^ represents a crucial advancement by providing a framework grounded in physiological principles for newborn adaptation. More precisely, it determines the appropriate timing for Umbilical Cord Clamping (UCC) based on physiological conditions [10], [11], [12], [6]. This physiology-based clamping approach has demonstrated benefits for the newborn compared to traditional time-based clamping [13], [14] and milking of the umbilical cord [15], primarily by improving the cardiovascular system and systemic and cerebral oxygenation [16], [13], [17], [18].

### 1.1 Motivation

Numerous studies in the literature have proposed guidelines for Neonatal Adaptation [19], [20], [21], [22]. However, few guidelines have focused on transitioning from time-based clinical practices at birth to practices centered on the physiological evolution of the patient, as highlighted in the INA guideline, proposed by the Division of Perinatal Pediatrics, Perinatology, and Neonatology at the National University of Colombia [10], [11], [12], [6]. This approach has shown promising results in patient outcomes, leading to its adoption by other countries such as Mexico since 2009 [23].

Due to the significant impact of the INA guidelines, developed by the Division of Perinatal Pediatrics, Perinatology, and Neonatology at the National University of Colombia, they serve as the foundational knowledge base for this study. While the INA guidelines represent a considerable advancement in neonatal care, effectively communicating and implementing these guidelines across diverse clinical settings remain challenging. This study aims to address this challenge through advanced knowledge representation techniques, which have been widely demonstrated in the literature to improve the communication and implementation of clinical guidelines [24], [25], [26], [27], [28]. This approach is particularly relevant in the neonatal domain, as evidenced by [29], one of the few works that partially represent a segment of the Neonatal Resuscitation protocol from the American Heart Association (AHA) [19] using formal protocols.

Furthermore, this study introduces a novel approach to the representation of the INA using statecharts, a method based on the principles of knowledge representation and engineering that can be easily extrapolated to computational simulators [30]. Statecharts, with their graphical nature, offer a clear and intuitive description of complex clinical processes, making them a very suitable tool for encoding medical guidelines. The choice of statecharts is motivated by their inherent properties of clarity, precision, and flexibility, essential for accurately representing clinically validated and widely accepted medical protocols.

### 1.2 Contributions

This study makes several significant contributions to the field of neonatal care and medical guideline representation, as outlined below:

**C1**. The novel application of statecharts for the representation of the INA guideline is introduced, offering a new perspective on knowledge representation techniques within medical guidelines. This marks a pioneering step in utilizing statecharts to enhance the clarity and accessibility of complex clinical protocols.
**C2**. The study uses a clinically validated flowchart from the INA guideline as the basis for the statechart model, ensuring that the representation is deeply rooted in evidence-based practices that have been shown to be effective in neonatal care. This methodological approach underscores the importance of providing representations of medical guidelines in clinical validation.
**C3**. By showcasing the reusability, adaptability, and scalability of statecharts, this study emphasizes their role as a flexible tool for illustrating the INA guideline in medical education. This approach facilitates the learning and implementation of complex clinical guidelines, addressing a crucial need in medical education for more accessible and adaptable educational resources.
**C4**. This work sets a precedent for the broader application of statecharts and similar knowledge representation techniques in healthcare protocols. This contribution is important as it opens new perspectives for the development and implementation of clinical guidelines in various healthcare settings, promoting better compliance, understanding and outcomes for patients.

Adopting a knowledge representation approach through statecharts, this study proposes a new model for INA guidelines, advocating the integration of such innovative methods in the representation of medical guidelines at large. The structured, clear, and adaptable framework offered by the statechart model supports the effective dissemination and implementation of the INA guideline, contributing to improved outcomes in neonatal care. This work aims to bridge the gap between clinical research and practice, ensuring the realization of the benefits of Physiologically-based Cord Clamping (PBCC) in clinical settings worldwide.

This study is structured as follows: Section 2 introduces related works concerning the representation of clinical protocols using formalisms. Section 3 details the proposed formal representation methodology, as well as the model assessment methodology. Section 4 presents the results of this research. Section 5 discusses the findings and outlines the limitations and future work. Finally, Section 6 concludes with the insights garnered from this study.

## 2 Related work

To the best of the authors' knowledge, research on the partial or complete representation of Neonatal Adaptation guidelines using formal mathematical representations is notably sparse, with only a handful of examples, such as the work by Wilson et al. (2006) [29], venturing into this domain. The scarcity of similar studies highlights the pioneering nature of the approach proposed in this study, positioning it at the forefront of innovative methodologies in the field. Consequently, this section aims to elucidate the application of formal models in the description of medical guidelines, a critical step in advancing the understanding and implementation of such frameworks. The realm of formal models in the representation of medical guidelines can be broadly divided into two distinct categories: *human-centered models* and *computer-centered models*.

In the realm of human-centered models, the outcome is a readily understandable graphical representation that encapsulates the sequences of actions within a clinical protocol. The main tool utilized in these human-centered models is the flowchart, which is highly valued for its straightforward interpretability. Various implementations of this methodology have been documented. For instance, Robinson et al. [31] introduced a *flowchart* for administering nefiracetam, a nootropic agent aimed at enhancing blood flow and boosting the morale and vigor of patients post-stroke. Similarly, the American Academy of Pediatrics (AAP) has crafted a structured protocol to address jaundice in healthy newborns [32]. Furthermore, Gehm et al. [33] have developed a formalized approach for the management of type 2 diabetes mellitus, a guideline established by the Dutch Association of General Practitioners.

Addressing a subject more closely aligned with this study, Sæther et al. [34] introduce a new resuscitation protocol for use in the delivery room, employing portable resuscitation units. This protocol is designed to assess the impact of Early Cord Clamping (ECC) on assisted vaginal deliveries of full-term and near-term infants. Nolan et al. [35] detail a *flowchart* for the neonatal resuscitation algorithm, synthesizing insights from seven systematic reviews, three scope reviews, and twelve evidence updates. The purpose of this *flowchart* is to foster consensus on various clinical procedures by improving the education and training of clinical professionals, covering practices such as suction in cases of clear and meconium-stained amniotic fluid, applying sustained inflations to begin positive pressure ventilation, and determining the initial oxygen concentrations for resuscitating premature and full-term newborns, among others. The final goal of these protocols is to improve the teaching and training of clinical professionals.

To structurally organize the information contained within clinical protocols and enhance the learning process, *human-centered models* have effectively evolved into *computer-centered models* [36], [37], [28], [38], [39]. This transition facilitates the systematic arrangement of clinical data, thereby improving educational methodologies in medical training.

In *computer-centered models*, the output is code interpreted by machines [40]. In particular, ten Teije et al. [28] used the medical planning language *Asbru* with the aim of minimizing the ambiguity and gaps often found in medical protocols, and assessed its viability through formal modeling and verification of the jaundice protocol. The efficacy of the *Asbru* language is further evidenced by Andreas Seyfang et al. [26], who integrated diagnosis and treatment strategies from the AAP for managing hyperbilirubinemia in newborns. Similarly, Méry and Singh [36] studied the language *Event B* to model and formally verify medical protocols. Böckmann et al. [41] and Johnson et al. [42] adopted a *Pathways*-based, model-driven methodology to translate clinical guideline narratives into a format interpretable by computers. However, the effectiveness of these models is dependent on the specific experiences and expectations of the institution. Addressing this challenge, Scheuerlein et al. [43] introduced an innovative method employing Business Process Modeling Notation (BPMN) and Tangible Business Process Modeling (TBPM), leveraging the BPMN standards, commonly used in business process management, to improve medical education, patient information, and quality management.

Barzdinsg et al. [44] introduced a specialized process modeling language for the medical domain named *Medmod*, which is essentially a detailed computational adaptation of Unified Modeling Language (UML) class diagrams for medical applications. According to the authors, UML diagrams effectively map out the sequence of actions in a medical procedure. Currently, graph theory has been applied to effectively encapsulate medical data. For example, Pham et al. [45] developed a *heterogeneous information graph* to group medical domain knowledge for patient health status prediction; Li et al. [46] designed a *knowledge graph* to distill medical insights from electronic medical records of knee osteoarthritis patients, which helps in decision making processes; similarly, Liu et al. [47] utilized the *knowledge graph* technique to explore and forecast potential links between the gut microbiota and mental health issues; and Huang et al. [48] established a *knowledge graph* to systematically represent the knowledge of Kawasaki disease, encompassing clinical guidelines, trials, pharmacological databases and scholarly articles. Furthermore, approaches such as *Workflows* (represented through graphs or timed automata) have been employed by researchers such as Gao et al. [49] and Wu et al. [50] to study, verify, and refine structured human interactions, thus enhancing precision within cyber-physical medical systems. In a related vein, Rahmaniheris et al. [51] applied communicating organ *state machines* to depict transitions between organ states, modeling changes in patient conditions during cardiac arrest scenarios.

Concerning state diagram models, the central formalism of this study, Guo et al. [52] have effectively utilized statecharts for collaborative interactions with medical professionals to delineate best-practice guidelines. Subsequently, these statechart models are transformed into timed automata, facilitating the application of formal verification tools. The adoption of statechart models in the medical field has grown considerably, attributed to their exceptional ability to succinctly summarize essential aspects of medical guidelines [53], [52], [54], [55], [24], [56].

## 3 Methods

To provide a comprehensive understanding of the fundamental modeling concepts applied in this work, the following topics are reviewed: *(i)* flowcharts of medical protocols; *(ii)* finite automata and their application in medicine; *(iii)* Statecharts; and *(iv)* Methodology of assessment of formal models. The examples in this section, while not related to the INA guideline, serve to illustrate the necessary concepts for this study.

### 3.1 Flowcharts of medical protocols

Flowcharts are commonly used to delineate medical protocols, condensing complex procedural information into an algorithmic format that is easy to understand. These visual tools facilitate the systematic presentation of step-by-step processes in medical practice, enhancing clarity and communication among healthcare professionals.

**Figure 1:**
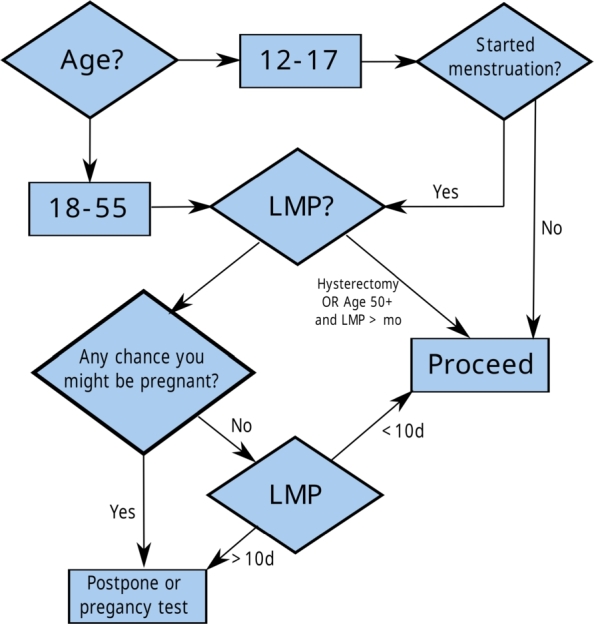
Flowchart of risk evaluation of patients with possible gestation for a diagnostic nuclear medicine examination (Taken from James and Warren-Forward [57]). The abbreviation LMP means *Last Menstrual Period*.

Example 1 The *flowchart* in Fig. 1 is a protocol to define the risk of a possible gestational state for a diagnostic nuclear medicine test [57].  □

The *flowchart* in Fig. 1 represents a consensus reached systematically among various medical experts using methodologies such as the *Delphi* technique [58]. Creating these flowcharts involves meticulous efforts by healthcare professionals, requiring multiple rounds of evaluation before formalizing them as protocols or guidelines.

When selecting a formal tool for modeling medical protocols within a *human-centered context*, it is crucial to evaluate the following features:

• *Versatility:* The tool should effectively model various medical guidelines and protocols in a comprehensible way.
• *Usability:* The tool should be user-friendly, facilitating the understanding of modeled medical guidelines and protocols.
• *Scalability:* The models should be easily adjustable, allowing progressive refinement of details within a medical procedure.
• *Moderate visual complexity:* The visual representation should be clear enough to be easily interpreted by a broad audience, including students, healthcare professionals, engineers, and others.

To incorporate these characteristics, finite automata and their *statechart* extension is selected as the modeling tools for this study.

### 3.2 Finite automata and their application in medicine

Finite automata are mathematical models used to represent sequences of events or states, often employed in various fields, including medicine, to model complex systems and processes.

Formally, a nondeterministic finite automaton, denoted by *G*, is defined by the following quintuple:

(1) $$G=(\Sigma ,Q,f,q_{0},Q_{m}).$$

Each component of the quintuple defined by Equation (1) is defined as follows: Σ represents the set of events, *Q* the set of states, $f:\Sigma \times Q\to 2^{Q}$ the partial state transition function, $q_{0}\in Q$ the initial state, and $Q_{m}\subseteq Q$ the set of marked states. This example illustrates the relationship between a *flowchart* and a finite automaton.

**Figure 2:**
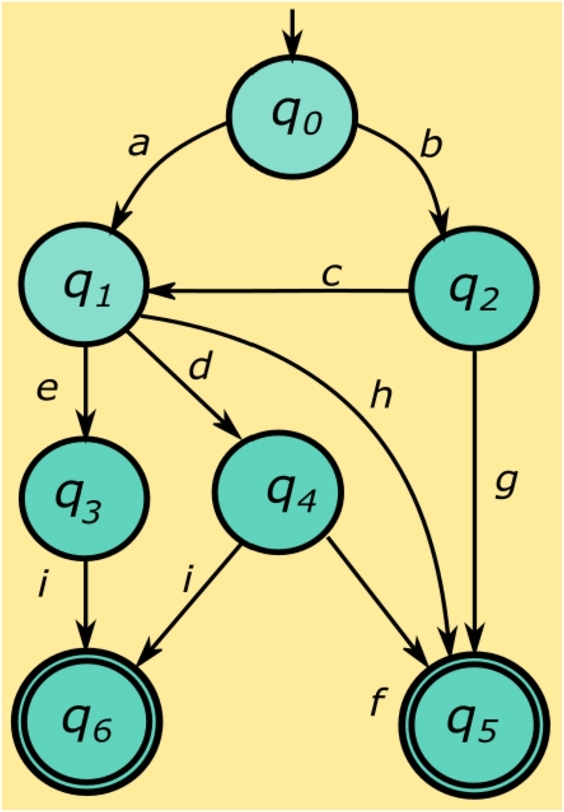
An automaton representing the *flowchart* of Fig. 1.

Example 2 Fig. 2 illustrates an automaton that models the medical *flow chart* shown in Fig. 1. The initial state of the automaton is denoted as $q_{0}$, symbolizing the commencement of a risk assessment test as detailed in Table 1. The automaton is defined by a set of states $Q=\{q_{0},\ldots ,q_{5}\}$, with the specific role of each state elaborated in Table 1. In particular, the states marked as $q_{5}$ and $q_{6}$ represent the completion of the procedure and the postponement of the procedure, respectively. The event set Σ={a,b,...,i} dictates the transitions between states, as outlined in Table 1 and the state transition diagram in Fig. 1. For example, event *a* classifies a woman within the age range of 18 to 55 years. The transition function *f* defines the permissible state transitions within the automaton based on procedural requirements. Mathematically, for automata, this is represented as $f(q_{1},e)=q_{3}$, translating medically to diagnosing a probable pregnancy state ($q_{3}$) based on the age range of the patient ($q_{1}$) and specific conditions (*e*). Table 1 further elucidates the medical interpretations aligned with the state and event labels of the automaton in Fig. 2.  □

**Table 1:** States and events of the automaton of Fig. 2.

(a) States of the automaton.	
	Definition
*q*_0_	start of the test
*q*_1_	established menstrual cycle
*q*_2_	uncertain menstrual cycle
*q*_3_	probable gestation
*q*_4_	improbable gestation
*q*_5_	the procedure is done
*q*_6_	the procedure is postponed


(b) Events of the automaton (medical indicators).	
*a*	18 < Age < 55 years
*b*	12 < Age < 17 years
*c*	Presents menstruation
*d*	Patient rules out possible gestation
*e*	The patient does NOT rule out possible pregnancy
*f*	Last menstrual period (LMP) < 10 days
*g*	She did not start menstruation
*h*	Hysterectomy or Age > 50 years
	or Last Menstrual Period > 12 months
*i*	Last Menstrual Period (LMP) > 10 days

A model of a medical procedure should exhibit a moderate level of visual complexity to enable easy verification and refinement by a team of experts. This requirement leads to the adoption of statechart representations, which are succinctly outlined below.

### 3.3 Statecharts

Statecharts, introduced by Harel [59], provide a hierarchical framework for representing finite automata. This study employs three essential components of this formalism: conditioned transitions, superstates, and orthogonal states.

**Figure 3:**
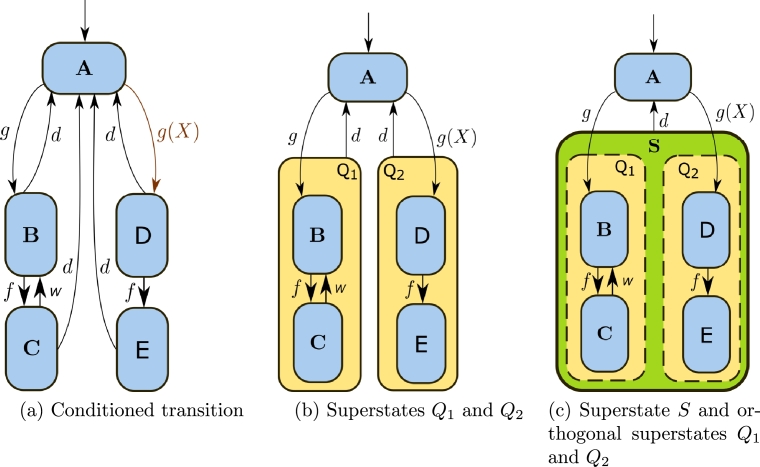
*Statechart* with conditioned transition, superstates, and orthogonal states.
▶ *Superstate:* Consider again the automaton of Fig. 3a. The occurrence of an event *d*, from one of states **B** or **C** (and **D** or **E**) produces the transition to state **A**. As illustrated in Fig. 3b, this can be visually refined by grouping the states **B** and **C** (resp. **D** and **E**) within a superstate, denoted by $Q_{1}$ (resp. $Q_{2}$). In this way, the two transitions from **B** to **A** (resp. **D** to **A**) and **C** to **A** (resp. **E** to **A**), are represented by a single transition marked by the event *d*, (which occurs similarly with the two transitions ranging from **D** to **A** and **E** to **A**), thus reducing the visual complexity in the graphic representation of automata.
  The semantics of the superstate $Q_{1}$ (resp. $Q_{2}$) is represented by an *exclusive-or* function of states **B** and **C** (resp. **D** and **E**). That is to say, if the model is in the superstate $Q_{1}$ (resp. $Q_{2}$), it must be in the state **B** or in the state **C** (resp. state **D** or state **E**), but not in both.
▶ *Orthogonal states:* The orthogonal states capture the co-occurrence of states. Consider now the automaton of Fig. 3b. The occurrence of the event *d*, from one of the superstates $Q_{1}$ or $Q_{2}$ implies the transition to the state **A**. As shown in Fig. 3c they have been grouped into a superstate denoted by **S** to further reduce the visual complexity. Notice that in the automaton of Fig. 3b there are two outgoing transitions from state **A**: the transition $A\overset{g}{\to }B$ (triggered by event *g*) and the conditioned transition $A\overset{g(X)}{\to }D$ (triggered by event *g* together with condition *X*). This fact brings about the possibility that the two transitions may occur concurrently.
  To correctly represent this behavior, the statechart formalism defines a feature called *orthogonal states*. The *orthogonal states* are represented by discontinuous rectangles within a superstate, which contains states that can be active simultaneously. For example, the superstate **S** of Fig. 3c includes the *orthogonal states* $Q_{1}$ and $Q_{2}$.
  The semantics of orthogonal states defines an *and* function of the states $Q_{1}$ and $Q_{2}$. Then, there exists the possibility of activating them simultaneously. Entry from a state to an orthogonal state such as $Q_{1}$ or $Q_{2}$ ensures the activation of at least one inner state. For example, the orthogonal state $Q_{1}$ (resp. $Q_{2}$) starts in the inner state **B** (resp. **D**) and the occurrence of event *f* changes to inner state **C** (resp. **E** inside $Q_{2}$). Notice that if states **B** and **D** are both active, the occurrence of event *f* simultaneously activates states **C** (inside $Q_{1}$) and **D** (inside $Q_{2}$).

▶ *Conditioned transition:* Consider the automaton of Fig. 3a. The transition  in the automaton of Fig. 3a is a *conditioned transition* which, according to the statechart formalism, implies that the automaton passes from state **A** to state **D** if and only if the event *g* occurs and the condition *X* is satisfied.

#### 3.3.1 Advantages of statechart models over traditional flowcharts

Statechart models provide numerous advantages over traditional flowcharts when representing clinical guidelines, offering improved clarity, scalability, and computational applicability. These characteristics make statecharts particularly effective for modeling complex and dynamic medical processes that demand precision and adaptability.

One of the key benefits of statechart models is their **hierarchical organization**, which includes states, superstates, and orthogonal states. This structure allows for a more comprehensive representation of intricate medical workflows. By grouping related processes within superstates, the visualization is simplified, and redundancy is minimized. The modular design of statecharts also enables the representation of nested processes and concurrent states, which is essential for depicting complex medical protocols [30], [54].

Unlike traditional flowcharts, statecharts incorporate **built-in memory**, which enables them to retain information about the current state of the system. This memory capability is particularly valuable for modeling medical workflows that depend on previous conditions or states. It enhances the accuracy of decision-making and tracking within clinical protocols, offering a level of precision that traditional flowcharts cannot match [52], [53].

Another significant advantage of statecharts is their ability to represent **parallel processes**. Using orthogonal states, statecharts can model multiple simultaneous activities, a feature that is crucial in medical scenarios where concurrent interventions, such as monitoring and resuscitation, are required. Traditional flowcharts, with their inherently linear structure, struggle to represent such parallelism without becoming overly complex [54], [55].

Statecharts also excel in depicting conditional transitions, allowing for the modeling of decision-making paths based on specific criteria. These conditioned transitions **reduce visual clutter** by only displaying paths relevant to certain conditions, improving both readability and precision [30], [52]. In contrast, traditional flowcharts often require numerous decision nodes, which can make them cumbersome and difficult to interpret.

**Scalability and flexibility** are additional strengths of statecharts. They can be progressively refined to incorporate more detailed subprocesses or integrated into larger systems as needed. This adaptability is particularly important for representing medical guidelines that may evolve or expand over time. Traditional flowcharts, on the other hand, lack modularity and become unwieldy when scaled up to include more complex information [30], [54].

From a computational perspective, statechart models are highly advantageous. They can be **easily translated into executable code** and integrated into decision support systems, enabling real-time simulations and automated validation of clinical protocols. Tools such as Stateflow [60], [61] allow direct conversion of statechart models into C code, making them well-suited for embedded systems and real-time applications [52]. Traditional flowcharts, however, often require extensive manual effort to adapt for computational use [62].

Finally, statecharts support **formal verification and validation method**s, ensuring that clinical guidelines meet safety and efficacy standards through rigorous testing [30], [52]. This process helps ensure logical consistency and completeness in state transitions, reducing errors and enhancing patient safety. Traditional flowcharts, lacking formal semantics, do not provide the same level of verification and validation [63].

### 3.4 Methodology of assessment of formal models

The methodology employed to assess the proposed formal model for the INA guidelines involved a comprehensive evaluation by an interdisciplinary panel. This panel, consisting of health and engineering professionals who are not involved in the research process, ensured an objective assessment. The assessment strategy aimed to validate the utility and applicability of statecharts in modeling medical guidelines, focusing on their potential to enhance educational outcomes and clinical practice.

#### 3.4.1 Interdisciplinary evaluation

An interdisciplinary evaluation approach is chosen to ensure the robustness of the model and applicability across various professional domains. This approach aligns with the findings of Cabana et al. (1999) who emphasize the importance of interdisciplinary collaboration in improving clinical guidelines implementation [64].

#### 3.4.2 Assessment criteria

The assessment focused on several criteria, including clarity, comprehensiveness, usability, and scalability. These criteria are chosen based on established frameworks in medical education and engineering best practices [65].

• **Clarity:** The model should be easily understandable to both medical and engineering professionals, facilitating interdisciplinary communication.
• **Comprehensiveness:** The model should cover all relevant aspects of the medical guideline it represents, ensuring no critical information is omitted.
• **Usability:** The model should be user-friendly and applicable in real-world settings, supporting both training and practice.
• **Scalability:** The model should be adaptable to various levels of detail and complexity, allowing for progressive refinement and expansion.

#### 3.4.3 Feedback and iteration

Feedback was gathered through structured surveys and interviews with participants from both the medical and engineering fields. This iterative process of feedback and refinement is supported by research in educational methodologies and model validation [66].

## 4 Results

This section introduces the statechart model of *flowchart* representing the INA guideline, developed by the Division of Perinatal Pediatrics, Perinatology and Neonatology of the National University of Colombia [10], [11], [12], [6].

### 4.1 Flowchart of the INA guideline

**Figure 4:**
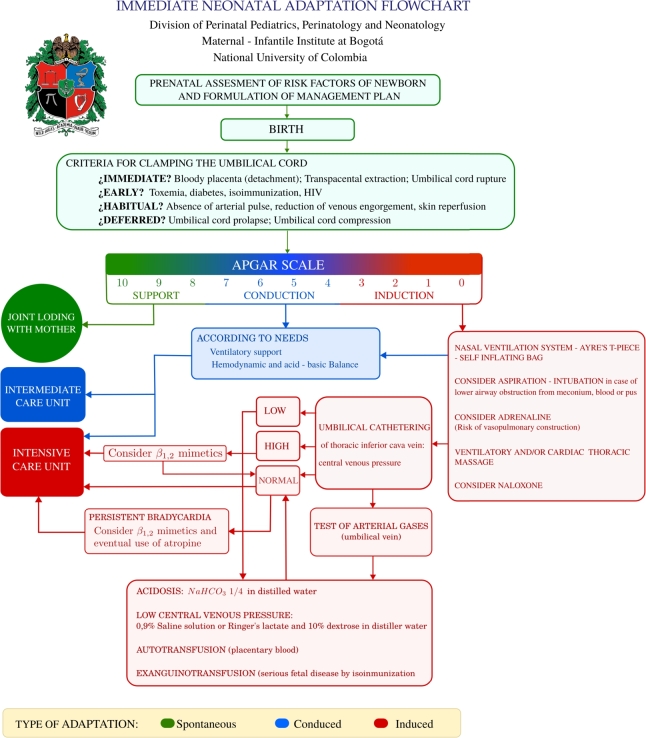
Translation and graphic adaptation of the original *flowchart* of the INA guideline of the DPN-UNC, which was originally published in Spanish [11].

Fig. 4 illustrates the *flowchart* for the INA guideline [10], [11], [12], [6]. This *flowchart* aims to detail the clinical procedures for adapting a newborn to its new life conditions.

This guideline advocates the application of the *Apgar* [67] and *Silverman–Andersen* [68] scales to assess a transition to extrauterine life of the newborn. The *Apgar* scale [67] is used to measure the vitality of the newborn, monitoring its progression at various intervals through a minute-to-minute evaluation during the neonatal adaptation phase. On the other hand, the *Silverman–Andersen* scale [68] is used to determine the presence and effort of ventilation. Furthermore, *flowchart* identifies four distinct types of UCC, categorized according to physiological criteria, making it a guideline based on PBCC.

Based on the signs and physiological conditions of the patient, the INA guideline categorizes the adaptation of the newborn to extrauterine life into three types: *spontaneous, conducted*, or *induced*. *Spontaneous adaptation* occurs when the essential conditions for the transition from fetal to neonatal life are naturally present and manifest. *Conducted adaptation* arises when these vital conditions exist but are in conflict or unstable, which requires careful management through medical intervention. *Induced adaptation* is required when these conditions are missing or nearly undetectable, thus needing to be initiated by specific medical procedures. The visual representation in Fig. 4 uses green blocks to denote *spontaneous neonatal adaptation*, blue blocks for *conducted adaptation*, and red blocks to describe the *induced adaptation* process [10], [11], [12], [6].

### 4.2 Statechart model of the INA guideline

**Figure 5:**
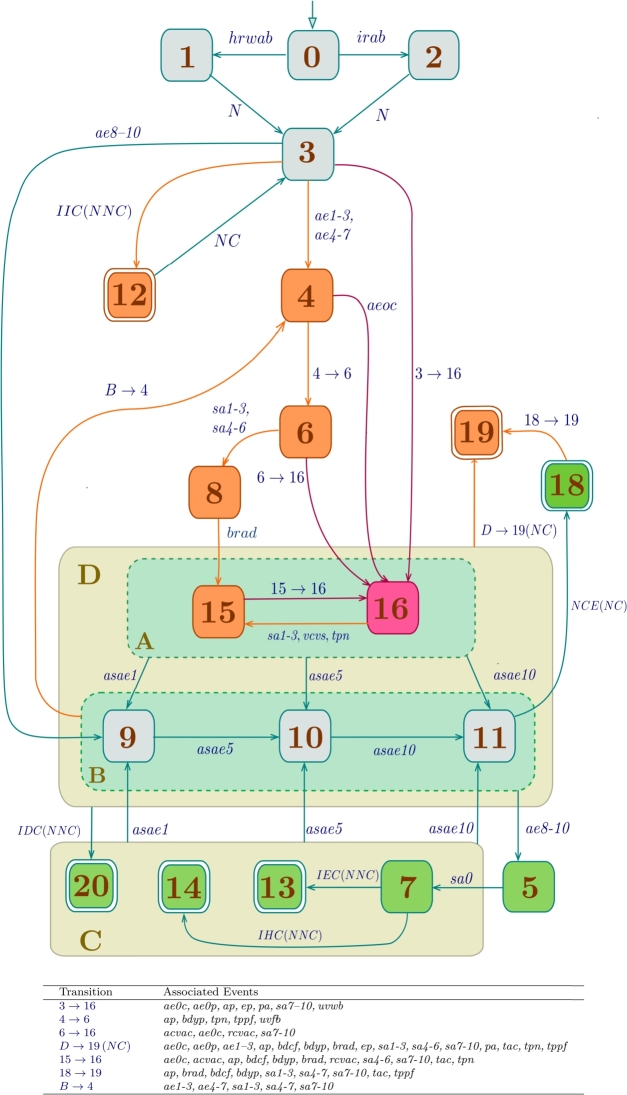
Statechart representing the INA.

The statechart model that corresponds to the *flowchart* of the INA guideline [10], [11], [12], [6], [69] is illustrated in Fig. 5. This model encapsulates detailed processes of spontaneous, conducted and induced neonatal adaptation as previously delineated.

#### 4.2.1 Methodology for developing the statechart model of the INA

The development of this model used an incremental refinement approach, beginning with a highly abstract model (*i.e.,* one with minimal detail) that underwent progressive refinement and correction through collaborative efforts between specialists in perinatology, neonatology, and engineering. It is crucial to emphasize that the creation of this model involved incorporating an enhanced level of detail, enriched by the clinical expertise and interpretative insights of the participants.

#### 4.2.2 Description of the statechart model for the INA

Consider the statechart of the INA as shown in Fig. 5. This model consists of 20 states (labeled from  to  and  to ), 36 events, and 4 superstates (labeled , , , and , detailed respectively in Table 2, Table 3, Table 4.

**Table 2:**
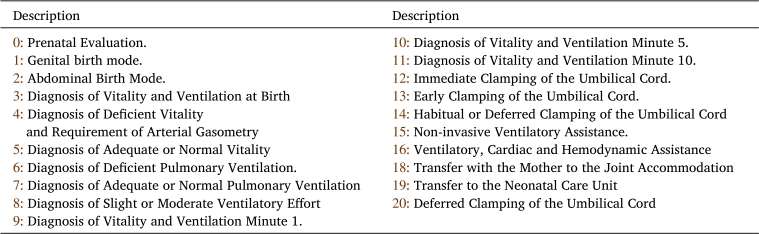
States of the INA statechart shown in Fig. 5.

**Table 3:**
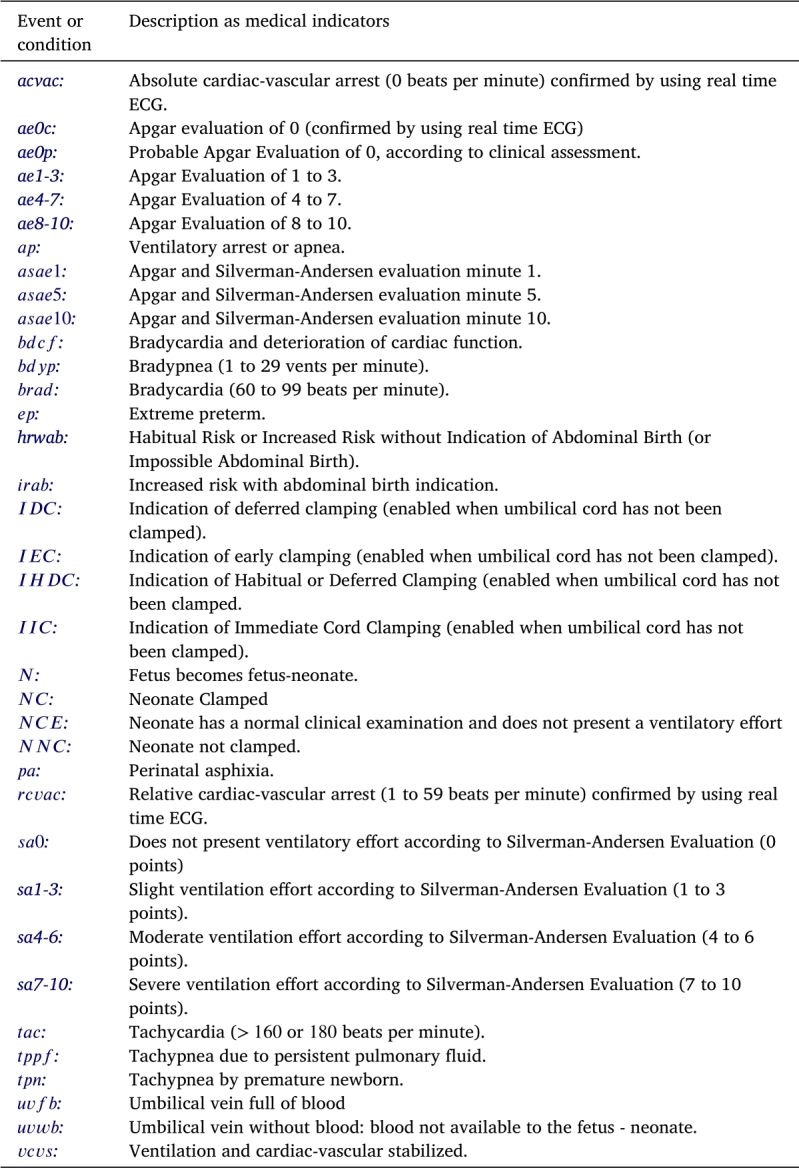
Events of the INA statechart shown in Fig. 5.

**Table 4:**
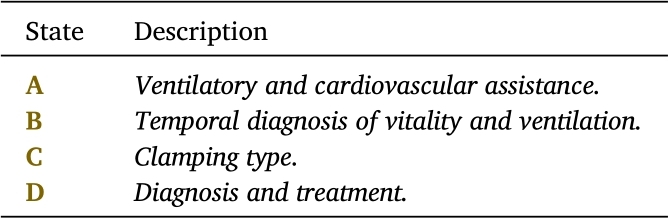
Superstates of the automaton of Fig. 5.

Observe two key attributes of this model. Firstly, every state, as outlined in Table 2, denotes either a diagnostic step or a procedural action required for the patient. Second, within the mathematical framework of automata, the term “event” is related to a clinical indicator –whether it is a vital sign, symptom, or patient characteristic as itemized in Table 3—which can be observed by a healthcare professional, meaning *an automaton event directly equates to a medical event*. An example provided below exemplifies this characteristic.

Example 3 Consider the transition  within the statechart shown in Fig. 5. Observe that the event  signifies a confirmed *Apgar* Evaluation of 0. Consequently, the automaton transitions from state 6 - where the newborn is identified with deficient vitality - to state 16, where the newborn receives ventilatory, cardiac, and hemodynamic support.  □

The states and transitions within the model are visually distinguished using color-coding to highlight the clinical status of the neonate. States shaded in gray represent normal conditions or diagnoses, indicating no immediate health concerns for the neonate. For instance, state 3 reflects the successful completion of the birth process for the newborn. States highlighted in orange, along with their corresponding incoming transitions, signify conditions that require medical intervention or careful monitoring of the progress of the neonate. State 16, marked in magenta along with its associated incoming transitions, represents a critical situation where the neonate requires ventilatory, cardiac, and hemodynamic support. Lastly, states shown in green indicate positive neonatal adaptation, allowing for umbilical cord clamping to proceed in accordance with the INA guideline.

To simplify the model, *conditioned transitions*, such as the transition  (refer to Fig. 5) are utilized. This transition is activated only when the event  (indicating early clamping) occurs, and the condition  (indicating the neonate has not been clamped) is met. Similarly, to prevent visual clutter, transitions involving multiple events are detailed in the table beneath the statechart in Fig. 5. For example, the symbol , labeling the transition from state  to state , signifies the transition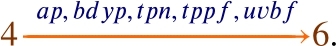 Let us now explore the different components of the model depicted in Fig. 5.

The *initial state* (labeled as  in Fig. 5) corresponds to the *prenatal evaluation*. In this state, prenatal control of the pregnant and evaluation of the fetus are carried out. Prenatal evaluation requires the presence of the mother and medical professional; interaction with the mother, father and family; clinical history; initial analysis of the case; and analysis of sequences.^2^

Table 2 describes the states of the INA statechart illustrated in Fig. 5. These *states* are classified into five groups: *(i)* mode of birth; *(ii)* diagnosis of vitality and pulmonary ventilation; *(iii)* type of clamping; *(iv)* marked states; and *(v)* superstates. Next, each group of states is discussed.

*(i).* *Mode of birth.* States  and  signify the mode of birth of the newborn. For the event  (*Habitual Risk or Increased Risk without Indication of Abdominal Birth*, refer to Table 3), no extra instruments are necessitated. Conversely, the event  (*Increased risk with abdominal birth indication*, as listed in Table 3) requires the use of instruments such as a vacuum cup, spatulas, forceps, and the provision for a cesarean section.
*(ii).* *Vitality and pulmonary ventilation diagnosis.* The states  reflect the current status of the newborn concerning its development, survival, and recovery capabilities from any imbalances incurred during gestation or the birthing process. The vitality assessment employs the *Apgar* scale [67], while the evaluation of ventilation utilizes a combination of the *Apgar* and *Silverman-Andersen* scales.
  State  is defined as *Diagnosis of Vitality and Ventilation at Birth*. This state encapsulates the vital conditions of the newborn at the moment of birth and tracks its progress through the first minute. States  depict the assessed conditions of the neonate regarding its vitality and ventilation capabilities. Specifically, states  evaluate the ventilation difficulties of the newborn. States , and  are designated for assessments conducted at the 1st, 5th, and 10th minutes post-birth, in alignment with the *Apgar* scale guidelines for assessing the physiological (or pathological) progression of the adjustment to neonatal life of the newborn.
*(iii).* *Clamping type*. The act of *UCC* involves occluding the umbilical arteries and veins to stop blood circulation. Based on the physiological classification introduced by Currea et al. [10], [11], [12], [6], there are four distinct clamping methods: *immediate, early, habitual,* and *deferred*. The states identified by  (refer to Table 2) specify the *clamping type* applied to the neonate, further elaborated below.
  ▶ State  represents *immediate clamping of the umbilical cord*, also known as *clamping at birth*. This procedure is recommended under any of the following circumstances: *(i) rupture of the umbilical cord; (ii) placental detachment leading to a bloodless umbilical vein; (iii) presence of a bloodless umbilical vein; and (iv) catastrophic placental bleeding.* The occurrence of any such condition triggers the conditioned transition  (refer to Fig. 5). As specified in Table 3, the event  denotes *Indication of Immediate Cord Clamping (ICC)*.
  ▶ State  is defined as *early clamping of the umbilical cord*. Recommended for neonates born to mothers with toxemia or diabetes to mitigate the risk of polycythemia, and in instances to limit the transmission of antibodies and viruses to the fetus, notably in cases of hemolytic disease due to maternal immunization against RhD antigen, myasthenia gravis, or maternal viral diseases like HIV and CMV. The occurrence of any aforementioned condition triggers the conditioned transition  as illustrated in Fig. 5. As detailed in Table 3, the event  signifies *Indication of ECC*.
  ▶ The state  signifies *habitual clamping of the umbilical cord*. This clamping method aims to achieve optimal pulmonary-systemic perfusion and facilitate the transition of the fetal circulatory system to a pattern suitable for extrauterine life. The completion of this physiological process is marked by the presence of conditions A1−A3 in the newborn, which typically manifest between 2 to 3 minutes (and up to 5 to 6 minutes) post-birth. The conditioned transition  shown in Fig. 5 indicates that conditions A1−A3 have been met. According to Table 3, the event  is indicative of *Indication of Habitual Cord Clamping (HCC)*.
  ▶ State  denotes the *deferred clamping of the umbilical cord*. This procedure is recommended in scenarios such as cord prolapse, breech birth, prolonged rupture of membranes, presence of circulars, bands, funicular knots, perinatal asphyxia, or when there is a deficiency in the vitality or ventilation capacities of the newborn, among other conditions. The occurrence of any of the mentioned conditions triggers the conditioned transition  as shown in Fig. 5, symbolizing the *Indication of Deferred Cord Clamping (DCC)* as detailed in Table 3.
  Remark 1 When any of the aforementioned conditions are present, the neonate typically attains the physiological states **A1-A3** in an extended period (generally exceeding 6 minutes post-birth) compared to the usual adaptation timeline. Therefore, in such clinical contexts, the practice of UCC is classified as *deferred*.
*(iv).* *Marked states.* This group of states, illustrated with a double line in Fig. 5, delineates critical junctures in the neonatal adaptation process. As previously mentioned, the states , , , and  specify the clamping method utilized. States  and  are described as follows:
  ▶ State  signifies that the neonate is ready for joint accommodation with the mother. At this stage, the mother receives hands-on instruction on the care of the newborn, fostering the normal growth and development of the newborn.
  ▶ State  denotes the necessity of transferring the neonate to a neonatal care unit, which could be either intermediate or intensive, based on the specific health needs of the neonate.
*(v).* *Superstates.* The medical interpretation of Superstates **A**, **B**, **C** and **D** is summarized in Table 4.
  In Fig. 5, it is observable that superstates  and  are orthogonal, implying that the neonate can simultaneously receive ventilatory support as the assessment of its vitality and ventilation occurs. The following sections provide a concise overview of these superstates.
  ▶ The superstate denoted by  (*Ventilatory and Cardiovascular Assistance*) covers states  and . State  is associated with *non-invasive ventilatory assistance*, signifying that the neonate does not require skin incision or the insertion of medical instruments or devices. Conversely, state  requires *invasive ventilatory assistance* and/or *hemodynamic support*, and/or *cardiac-vascular massage*. This condition requires performing an arterial gasometry test, which can be performed by puncture or, as an alternative, catheterization if needed.
  ▶ The superstate denoted by  (*Temporal Diagnosis of Vitality and Ventilation*) includes states , , and , which correspond to the assessments of vitality and ventilation at the 1st, 5th, and 10th minutes, respectively. Within this superstate, evaluations of vitality and ventilation are conducted utilizing the *Apgar* scale for vitality and the *Silverman-Andersen* scale for ventilation.
  ▶ The superstate identified by  (*Clamping Type*) encompasses the states related to the clamping method of the umbilical cord: *diagnosis of adequate or normal ventilation* (state ); *early clamping of the umbilical cord* (state ); *habitual clamping of the umbilical cord* (state ); and *deferred clamping of the umbilical cord* (state ), all of which have been previously described.
  ▶ The superstate denoted by  (*Vitality and Ventilation Medical Assistance*) groups the orthogonal superstates  and  outlined previously. This configuration suggests that the neonate can simultaneously undergo ventilatory support or cardiovascular massage in conjunction with the ongoing assessment of its vitality and ventilation.

It is crucial to emphasize that the hierarchical nature of statecharts allows for an enhanced level of detail in medical guidelines like the INA. Take, for example, state , which pertains to *Ventilatory, Cardiac, and Hemodynamic Assistance*. This particular state represents a complex clinical procedure that requires its own dedicated model.

### 4.3 Expert medical assessment of the proposed model

In this section, the results of the assessment of the proposed formal model for the INA guidelines are presented. This evaluation was conducted by a scientific committee comprising medical and engineering professionals who are not involved in the research process, ensuring the objectivity of the evaluation. The feedback received on this model is discussed, and the application of statecharts in modeling medical guidelines is assessed.

It is noteworthy that the Statecharts facilitated the creation of a universal visual language comprehensible across various professional disciplines. Detailed here is the feedback collected from 21 evaluators, including 3 engineering students, 4 pediatrics residents, and 14 fourth-year medical students, regarding the INA model showcased in this study.

**Figure 6:**
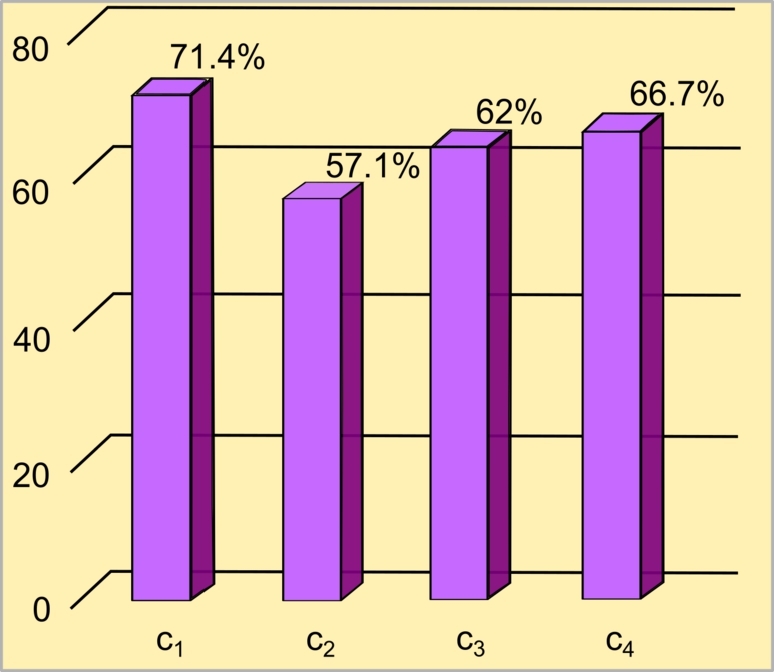
Results of validation by health professionals.

To evaluate the potential and usability of the statechart model of the INA shown in Fig. 6, opinions were solicited from 18 medical students (4 graduate and 14 undergraduate) regarding the following satisfaction criteria:

$c_{1}$: *Effectiveness of the mathematical, graphic and clinical language used.*
$c_{2}$: *Usability of the model in*Fig. 5*with respect to conventional representations.*
$c_{3}$: *Minimum educational level to use the model in*Fig. 5*.*
$c_{4}$: *Knowledge barriers that prevent the understanding of the content.*

Fig. 6 illustrates the satisfaction scores for the criteria evaluated ($c_{1}-c_{4}$) as rated by the participants. In relation to criterion $c_{1}$, as shown in Fig. 6, 71. 4% of the respondents found the mathematical, graphical and clinical language utilized in this representation to be clear, concrete, and applicable across various domains of knowledge. For the criterion $c_{2}$, as shown in Fig. 6, 57.1% believed that, compared to traditional methods of presentation of clinical information (such as flow charts or text), it serves as an intuitive tool that accurately conveys the information *flowchart* in Fig. 4, facilitating a straightforward and rapid comprehension of the immediate process of neonatal adaptation. They expressed a desire to be exposed to this tool earlier during their education. Regarding the criterion $c_{3}$, as indicated in Fig. 6, 62% thought that people with at least a level of secondary education could basically understand the process of INA, while 38% considered that a deeper understanding requires at least the first two years of medical training. Finally, regarding the criterion $c_{4}$, as shown in Fig. 6, 66.7% of the participants considered that there are no significant barriers in the transmission of knowledge or instruction that makes it difficult to understand the model.

**Figure 7:**
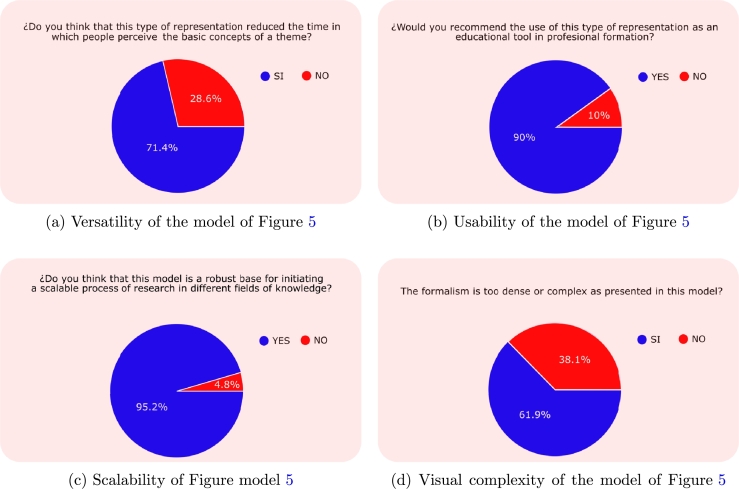
Characteristics of the model of Fig. 5 to be used in professional training.

Fig. 7 displays the evaluation of the four essential characteristics for a formal modeling tool of medical protocols, as introduced in the section on flowcharts of medical protocols. As shown in Fig. 7a, 71.4% of respondents believe that this representation method offers sufficient versatility to expedite the understanding of a fundamental concept of the subject. Furthermore, as shown in Fig. 7b, concerning the usability of the model, 90% of participants would endorse this representation form as a pedagogical aid in their professional education. As indicated in Fig. 7c, a notable 95.2% view this type of model as a solid foundation for launching scalable research initiatives across various knowledge domains. Finally, Fig. 7d reveals that 61.9% of the participants regard the representation as an effective approximation, considering the informational density of the diagram and the inherent complexity of human physiological processes, suggesting that the model facilitates straightforward extensions.

The following section shares some of the feedback from participants on their evaluation of the INA model introduced in this study.

#### 4.3.1 Healthcare professionals

▶ *The creation of a model that represents a routine procedure in a simple way for understanding serves as a basis for translating different interdisciplinary procedures. In the future, this will allow the posing of new and more complex models that include details from different areas of knowledge. However, visual complexity must be further reduced, bearing in mind that in the future the model will contain even more information.*
▶ *I would use this diagram since I find it very useful. This type of representation allows one to organize and document large amounts of clinical information efficiently.*
▶ *I consider that this type of representation can be a potential basis for initiating future research processes in different fields of knowledge. However, it is a challenge and it seems appropriate to start taking risks. In my opinion this is a significant advance, which still has aspects on the graphical interface to improve.*

#### 4.3.2 Engineering professionals

▶ *The development of this model allows the conception of tools for the presentation of medical guides and with different health training, including, of course, patients. This also allows the development of simulators with medical training scenarios.*
▶ *The development of models like the one presented here can be scalable to make diagrams of this type in other medical, biological or engineering processes. In addition, its greatest advantage is the possibility it offers to redirect events and make the process less “linear” and based on the behavior of the newborn.*
▶ *The abstraction of a biological model that considers as many possible scenarios in a risk situation for a newborn, converts or adapts the evaluation method mechanically without losing the notion of the evolution of the newborn.*

**Figure 8:**
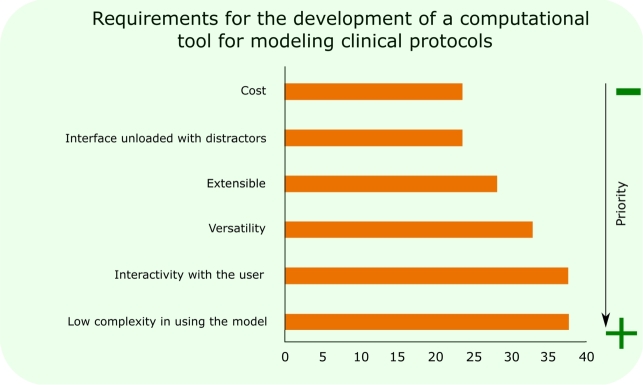
Design requirements for the development of a computational tool for modeling clinical protocols.

Considering the focus on creating computational tools to enhance health training processes, an assessment was conducted on the desired features of a computer-based educational tool. Fig. 8 displays the prioritization of these characteristics, ranked from lowest to highest importance according to participant feedback. As illustrated in Fig. 8, 38.1% of participants identified *low complexity in model usage* as the most crucial aspect, whereas *a distraction-free interface and cost* were considered the least important. In particular, *cost* is not considered a high priority factor in the choice of a computational tool for medical education and training, according to the feedback of the participants. This insight provides a direction for future enhancements to the model discussed in this study.

## 5 Discussion

The incorporation of statecharts in modeling the INA guideline demonstrates the effectiveness of visual formalism in simplifying and accurately outlining the framework of the guideline. This method notably reduces visual clutter by using superstates and conditioned transitions for state changes under specific circumstances, such as when the umbilical cord has not been clamped (*NNC*). This enhancement improves the clarity of the model and ensures that each state and transition is linked to clinical significance.

For a clearer understanding of the functionality of the proposed model, an example is presented to interpret its logic. According to Table 3, the condition  signifies that the umbilical cord remains unclamped. Therefore, conditioned transitions like  are triggered only if the umbilical cord has yet to be clamped. Medically speaking, the act of clamping and subsequently cutting the umbilical cord is irreversible, leading the neonate to the  condition where the cord is considered occluded. The absence of conditioned transitions would necessitate incorporating medically irrelevant states into the model, complicating its comprehension. This exemplifies the benefits of employing the statechart formalism.

The mathematical model devised through this study opens the way for future progress in understanding and implementing resuscitation algorithms more effectively. The structured and clear visual layout of this model establishes the foundation for computational tools aimed at aiding medical professionals. These tools can assess the effectiveness and application of neonatal resuscitation, allowing for adjustments based on the latest medical research or specific clinical protocols.

It is important to highlight that the results presented in this study are derived from a thorough evaluation involving an interdisciplinary panel consisting of healthcare and engineering professionals, along with medical students. The feedback indicated a high level of satisfaction with the *versatility, usability, scalability,* and *moderate visual complexity* of the model. To validate the responses, it is ensured that the respondents had relevant backgrounds and experience in dealing with formal models and medical guidelines. Most respondents had experience in neonatal care, clinical protocols, or engineering applications in healthcare, forming a solid basis for their evaluations. This diverse yet specialized panel ensured that the feedback is both comprehensive and contextually relevant.

### 5.1 Limitations

This study acknowledges potential biases and threats to validity. The main sources of bias include the subjective nature of self-reported data and the possibility of selection bias due to the backgrounds of the respondents in medical and engineering fields. To mitigate these biases, a robust evaluation framework was employed, ensuring that a diverse panel of evaluators was included [70]. Additionally, triangulation was used by combining multiple data sources and methods, which enhanced the reliability and validity of the findings. This approach ensured consistency across different scenarios, providing greater confidence in the robustness of the conclusions [71].

The model presented in this study offers a pioneering approach to representing the INA guideline, yet it is based on a flowchart developed within a specific local context (Colombia), reflecting regionally proposed practices. However, the formal representation introduced here was designed to be flexible, allowing for easy adaptation to different local practices or the development of a more generalized framework for neonatal care. It is important to note that neither the goal of the model proposed in this study nor the original flowchart is to quantify the certainty of the evidence surrounding individual resuscitation steps. To achieve this, a more thorough verification of the content is required, following practices recommended by the AHA [72].

While the model visualizes procedural flow and decision points effectively, it does not take into account the nuances of clinical judgment and individualized patient care. Future work should aim to incorporate these aspects, potentially through integrating the model with real-time data and decision support systems. Additionally, comprehensive validation in clinical settings is necessary to ensure alignment with current medical practices and seamless integration into existing protocols. This step would help identify potential barriers during implementation and enhance the model utility as a training and decision-support tool.

### 5.2 Future work

The next phase of this work aims to enhance the comprehensiveness of the model by leveraging the hierarchical capabilities of statecharts. Additionally, a transition to a computational implementation is planned, which could offer a more dynamic and interactive exploration of the guideline. Considering the complexity involved in clinical decisions, where factors such as Apgar scores, gestational age, and resuscitation guidelines play a crucial role, a computational tool with modular capabilities could significantly impact the adoption and utility of these representations. Future research may also explore analyzing historical resuscitation data to assess compliance and iteratively explore potential associations between resuscitation steps and patient outcomes, systematically refining the initial flowchart. The information of the formalism presented in this study will be enriched using current techniques of computational analysis such as those described in [73].

Expanding the applicability of this model to additional neonatal and pediatric care protocols represents a promising area for further exploration. The hierarchical and flexible design of statecharts allows this approach to be extended to other clinical protocols, including those used in neonatal intensive care, monitoring practices for premature infants, and emergency pediatric interventions. Applying this model to diverse aspects of neonatal and pediatric care can enable future research to identify shared patterns and enhance the implementation of guidelines across a wide range of clinical contexts.

In addition, studying the incorporation of artificial intelligence (AI) into the statechart model could offer predictive analytics and personalized recommendations for neonatal care, as demonstrated in other medical contexts [74], [75]. This integration would facilitate ongoing learning and adjustment of the model using real-world data, thereby enhancing its applicability and precision in clinical environments.

## 6 Conclusions

This study introduces a statechart model for the INA guideline, employing these models for the systematic exploration of various clinical scenarios. This approach strengthens the learning process for students, facilitating early and progressive interaction with knowledge at the outset of their professional careers. This not only enhances the potential for better outcomes but also provides ample time for concept assimilation. The model serves as a supportive tool for teaching and training in perinatology and neonatology.

The state diagram model proposed here offers a universal visual language, allowing interaction between specialists from various fields to develop models of medical procedures. This visual representation more clearly identifies patient states, criteria, and clinical indicators assessed during medical procedures, suggesting the potential for progressively detailed information within a medical guideline. Feedback generally reflected satisfaction with the characteristics of the model: *Versatility, Usability, Scalability,* and *Moderate visual complexity*, as proposed for a formal tool in the modeling of medical guidelines.

However, utilizing visual formalisms to represent medical procedures may risk students making decisions based only on the alternatives of the model. Therefore, it is crucial that the educational process fosters an understanding that these tools are merely guides and support in student development. Finally, the decision-making and application of the criteria should be performed by healthcare professionals based on their knowledge, medical evidence, and the clinical and evolutionary conditions of the patients.

Looking ahead, this study lays the groundwork for future research that could accelerate not only studies in the field but also the educational progression of health professionals. While this study focuses on neonatology, its methodologies and findings are applicable to other medical domains, promising widespread implications for healthcare education and practice.

As demonstrated, the integration of statecharts into the representation of medical guidelines marks a pioneering approach within the field. This not only enhances the comprehension and application of complex clinical guidelines but also encourages multidisciplinary collaboration, bridging gaps between theoretical knowledge and practical application in medical education and training. The potential of this model to improve clinical outcomes and educational efficiency invites further exploration and adaptation in broader medical contexts.

## Acronyms

**AAP**: American Academy of Pediatrics
**AHA**: American Heart Association
**BPMN**: Business Process Modeling Notation
**DCC**: Deferred Cord Clamping
**ECC**: Early Cord Clamping
**HCC**: Habitual Cord Clamping
**ICC**: Immediate Cord Clamping
**INA**: Immediate Neonatal Adaptation
**PBCC**: Physiologically-based Cord Clamping
**TBPM**: Tangible Business Process Modeling
**UCC**: Umbilical Cord Clamping
**UML**: Unified Modeling Language

## Ethics and consent

In this paper, a mathematical model of an existing medical guideline is developed. Specifically, a novel mathematical representation for the Immediate Neonatal Adaptation Guideline is proposed. No clinical or medical interventions, pharmaceutical substances, or patient records or clinical data are involved in this study.

The unique role of the participants in this study was the evaluation of various features of the proposed mathematical model. Accordingly, no sensitive data related to participants are included in the study, and no interaction with personal or medical information was required. In no way is any potential physical, emotional, psychological, or social harm posed to participants. Furthermore, the study does not involve the manipulation of clinical, social, or economic conditions of participants. All the participants were adults.

All participants were informed that, upon completion and submission of the study survey, their consent would be assumed and their anonymized responses would be considered as part of the study.

## CRediT authorship contribution statement

**Edgar Hernando Sepúlveda-Oviedo:** Writing – review & editing, Writing – original draft, Visualization, Validation, Supervision, Software, Resources, Project administration, Methodology, Investigation, Funding acquisition, Formal analysis, Data curation, Conceptualization. **Leonardo Enrique Bermeo Clavijo:** Writing – review & editing, Writing – original draft, Visualization, Validation, Supervision, Software, Resources, Project administration, Methodology, Investigation, Funding acquisition, Formal analysis, Data curation, Conceptualization. **Luis Carlos Méndez–Córdoba:** Writing – review & editing, Writing – original draft, Visualization, Validation, Supervision, Software, Resources, Project administration, Methodology, Investigation, Funding acquisition, Formal analysis, Data curation, Conceptualization.

## Declaration of Competing Interest

The authors declare that they have no known competing financial interests or personal relationships that could have appeared to influence the work reported in this paper.

## Data Availability

The data that support the findings of this study are available upon request from the corresponding author.

## Footnotes

1 The medical school of this division of the university has also developed the Kangaroo Mother Care, which has scientifically proven benefits for preterm newborns and has been adopted by many countries [8], [9].

2 This analysis includes the following aspects: prenatal diagnosis, probable interventions, functional organization of the medical team for the INA and also the verification of the environment, equipment and medicines for the INA.
